# Fenretinide-induced apoptosis of Huh-7 hepatocellular carcinoma is retinoic acid receptor β dependent

**DOI:** 10.1186/1471-2407-7-236

**Published:** 2007-12-31

**Authors:** Pengli Bu, Yu-Jui Yvonne Wan

**Affiliations:** 1 Department of Pharmacology, Toxicology and Therapeutics, University of Kansas Medical Center, 3901 Rainbow Boulevard, Kansas City, KS, 66160, USA

## Abstract

**Background:**

Retinoids are used to treat several types of cancer; however, their effects on liver cancer have not been fully characterized. To investigate the therapeutic potential of retinoids on hepatocellular carcinoma (HCC), the present study evaluates the apoptotic effect of a panel of natural and synthetic retinoids in three human HCC cell lines as well as explores the underlying mechanisms.

**Methods:**

Apoptosis was determined by caspase-3 cleavage using western blot, DNA double-strand breaks using TUNEL assay, and phosphatidylserine translocation using flow cytometry analysis. Gene expression of nuclear receptors was assessed by real-time PCR. Transactivation assay and chromatin immunoprecipitation (ChIP) were conducted to evaluate the activation of RXRα/RARβ pathway by fenretinide. Knockdown of RARβ mRNA expression was achieved by siRNA transfection.

**Results:**

Our data revealed that fenretinide effectively induces apoptosis in Huh-7 and Hep3B cells. Gene expression analysis of nuclear receptors revealed that the basal and inducibility of retinoic acid receptor β (RARβ) expression positively correlate with the susceptibility of HCC cells to fenretinide treatment. Furthermore, fenretinide transactivates the RXRα/RARβ-mediated pathway and directly increases the transcriptional activity of RARβ. Knockdown of RARβ mRNA expression significantly impairs fenretinide-induced apoptosis in Huh-7 cells.

**Conclusion:**

Our findings reveal that endogenous expression of retinoids receptor RARβ gene determines the susceptibility of HCC cells to fenretinide-induced apoptosis. Our results also demonstrate fenretinide directly activates RARβ and induces apoptosis in Huh-7 cells in a RARβ-dependent manner. These findings suggest a novel role of RARβ as a tumor suppressor by mediating the signals of certain chemotherapeutic agents.

## Background

Hepatocellular carcinoma (HCC), the primary malignancy of the liver, is the third most common cause of cancer-related mortality worldwide [1]. HCC is highly resistant to available chemotherapy, resulting in a 5-year relative survival rate of less than 7% [2]. Thus, discovery of new and effective therapies against HCC is much needed.

Retinoids, the natural and synthetic derivates of vitamin A, has a long history in clinical application in addition to its roles as an essential nutrient. Historically, Egyptians used roasted ox liver, which is rich in vitamin A, to treat night blindness. Nowadays, physicians prescribe drugs containing retinoids to treat dermatological disorders and leukemia. Moreover, data from experimental animal models and preclinical, epidemiological, and clinical studies suggest that retinoids may also have chemopreventive and anticancer effect. The best example of retinoid anticancer effect is the retinoic acid (RA) differentiation therapy for acute promyelocytic leukemia (APL) [3]. The use of RA has changed the clinical course of APL from a highly lethal to a curable leukemia, therefore establishing the prototype of retinoid-based therapies and the rationale for the use of retinoids in the treatment and prevention of cancer [4]. In addition, retinoids have been used either alone or in combination with other chemotherapeutic agents to treat other types of cancer and precancerous lesions. The anti-proliferative effect of tamoxifen is synergistically enhanced when used in combination with retinoids [5].

Retinoids also show promising effects in adjuvant therapy for HCC [6]. However, the therapeutic potentials of retinoids against HCC have not been extensively investigated. In the present study, we initiated a comprehensive screening including most commercially available retinoids on three widely used human HCC cell lines for apoptosis induction. Agree with previous studies [7,8], we found that fenretinide (N-[4-hydroxyphenyl] retinamide or 4HPR) induces apoptosis in Hep3B cells. In addition, we found that fenretinide also effectively induces apoptosis in Huh-7 cells. In contrast, HepG2 cells are resistant to fenretinide treatment. To elucidate the mechanisms underlying the observed differential susceptibility, gene expression analysis of twelve nuclear receptor genes were assessed by real-time PCR. Our data strongly suggest that the susceptibility of HCC cells to fenretinide treatment is determined by the basal and the induced expression level of RARβ. Furthermore, we showed that fenretinide directly activates RARβ in Huh-7 cells. Finally, the RARβ-deficient Huh-7 cells exhibited marked reduction of fenretinide-induced apoptosis. Based on these findings, we conclude that, in Huh-7 cells, fenretinide directly activates RARβ and induces apoptosis in a RARβ-dependent manner.

## Methods

### Reagents

The retinoids used in this study are grouped into three categories: (1) carotenoids including β-carotene, lycopene, and lutein; (2) classic retinoids including all-trans retinol palmitate, retinol acetate, 9-cis retinaldehyde, 13-cis retinol, 13-cis retinaldehyde, 13-cis retinoic acid, and fenretinide; (3) receptor-specific retinoids including all-trans retinoic acid (ligand for RAR), 9-cis retinoic acid (ligand for both RAR and RXR), and TTNPB (4-(E-2-[5,6,7,8-tet-rahydro-5,5,8,8-tetramethyl-2-naphthalenyl]-1-propenyl) benzoic acid) (ligand for RAR). β-Carotene, lycopene, all-trans retinol palmitate, 9-cis retinaldehyde, 13-cis retinol, fenretinide, all-trans retinoic acid, 9-cis retinoic acid, and TTNPB were purchased from Sigma-Aldrich (St. Louis, MO). Lutein was purchased from US Biological (Swampscott, MA). Retinol acetate and 13-cis retinaldehyde were purchased from Toronto Research Chemicals (North York, Canada). 13-cis retinoic acid was purchased from BIOMOL (Plymouth Meeting, PA). Retinoids were dissolved in dimethyl sulfoxide (DMSO) at 10 mM as the stock solution and stored at -80°C. Retinoids were diluted with serum-free medium to a 10 μM final concentration immediately before use. The final concentration of DMSO in the culture medium was 0.1% in all treatments. Because retinoids are light sensitive, all retinoid treatments were conducted under dim light.

### Cell culture

Huh-7 cells were cultured in Dulbecco's Modification of Eagle's Medium and HepG2 and Hep3B cells were cultured in Minimum Essential Medium (Mediatech, Herndon, VA). The media were supplemented with 10% fetal calf serum (FBS) (Atlanta Biologicals, Lawrenceville, GA). Cells were cultured at 37°C in 5% CO_2 _atmosphere with a relative humidity of 95%. Cells were plated with approximately 1 × 10^6 ^cells per T-25 flask and cultured overnight. The next morning cells were rinsed with PBS to remove FBS and incubated with individual retinoids (10 μM) in serum-free media for three days or as otherwise indicated. Fresh medium containing individual retinoids was provided every 24 hours. Cell viability was determined by trypan blue exclusion counting with a hemocytometer. Every sample was counted in triplicates.

### Immunoblotting and antibodies

Detached cells from individual retinoid treatments were collected every 24 hours and combined at the end of the treatment. Cells were lysed with lysis buffer (50 mM Tris·Cl pH 7.4, 150 mM NaCl, 2 mM EDTA, 0.1% SDS, 1% (V/V) NP-40 with protease and phosphatase inhibitors (Pierce, Rockford, IL)). Equal amounts of lysates (50 μg total protein) were run on SDS-PAGE and electroblotted onto PVDF membrane (Bio-Rad, Hercules, CA). The membranes were first incubated with PBS supplemented with 0.1% Tween 20 and 5% nonfat dry milk (PBST-milk) for 1 hour at room temperature to block nonspecific binding sites. Immunostaining was performed by incubating the membranes with primary antibodies for caspase-3 (Cell Signaling, Beverly, MA) or β-actin (Santa Cruz, Santa Cruz, CA) in PBST-milk overnight at 4°C. After three washes in PBST, membranes were incubated with the appropriate horseradish peroxidase-conjugated secondary antibodies for 1 hour in PBST-milk followed by three washes. Signal was detected using the ECL system SuperSignal West Pico Chemiluminescent Substrates (Pierce, Rockford, IL).

### Terminal deoxynucleotidyl transferase dUTP nick end labeling (TUNEL) assay

Cells (5 × 10^4^) were plated into chamber slides (BD, Franklin Lakes, NJ) in medium supplemented with 10% FBS and cultured overnight to attach. The next morning, cells were washed with PBS and incubated with fenretinide (10 μM) in serum-free medium for 24 hours followed by TUNEL staining using an *in situ *cell death detection kit (Roche Applied Science, Indianapolis, IN) according to the manufacturer's instruction.

### Flow cytometry

Cells (1 × 10^6^) were plated into T-25 flasks in medium supplemented with 10% FBS and cultured overnight to attach. The next morning, cells were washed with PBS and incubated with fenretinide (10 μM) for 24, 48, or 72 hours. Medium containing fresh fenretinide were provided every 24 hours. Attached cells were collected at each time point and processed for Annexin V-FITC and propidium iodide double staining using Annexin V-FITC Apoptosis Detection kit (BD Biosciences, San Jose, CA) according to the manufacturer's instruction. Samples were then analyzed for Annexin V-FITC positive cells on a Fluorescence Activated Cell Sorter (FACS) (BD Biosciences, San Jose, CA).

### Total RNA preparation

Total RNA was extracted with TRIzol reagent (Invitrogen, Carlsbad, CA) according to the manufacturer's instruction. RNA was quantified and assessed for purity on a UV spectrophotometer.

### Reverse transcription and real-time PCR

Total RNA (1 μg) was reverse-transcribed with oligo (dT) primer and M-MLVRT reverse transcriptase (Invitrogen, Carlsbad, CA) for cDNA synthesis. cDNA corresponding to 32 ng total RNA was used as the template in a 20 μl real-time PCR reaction with the ABI TaqMan Universal PCR Master Mix (Applied Biosystems, Foster City, CA) and the appropriate primer pair and Taqman probe. All primer pairs and Taqman probes were designed with Primer Express software v2.0 (Table 1). Real-time PCR was conducted using the ABI Prism 7300 real-time PCR system (Applied Biosystems, Foster City, CA). The quantification analysis for target gene expression was performed using the relative quantification comparative CT method [9].

**Table 1:** Real-time PCR primers and probes used in this study

**Target Gene**	**Gene Bank Acession NO**.		**Primer Sequence (5'-3') Sequences are shown for forward (F) and reverse (R) primers**	**Probe Sequence (5'-3')with modification of 5' FAM/3' BHQ1**
hRARα	NM_000964	F	GACAAGTCCTCAGGCTACCACTATG	CTGCAAGGGCTTCTTCCGCCG
		R	GTACACCATGTTCTTCTGGATGCT	
hRARβ	NM_000965	F	TCTCAGTGCCATCTGCTTAATCTG	CCAGGACCTTGAGGAACCGACAAAAG
		R	CCAGCAATGGTTCTTGTAGCTTATC	
hRARγ	NM_000966	F	GCTGCAAGGGCTTCTTTCG	CGAAGCATCCAGAAGAACATGGTGTAC
		R	CAGTTTTTGTCGCGGTGACA	
hRXRα	NM_002957	F	TCCTTGGAGGCCTACTGCAA	CAGCCGGGAAGGTTCGCTAAGC
		R	GCATTTGAGCCCGATGGA	
hRXRβ	NM_021976	F	AGCAGCAGGGACGGTTTG	AAGCTGCTGCTACGTCTTCCTGCCC
		R	GCTCTAGACACTTAAGGCCAATGG	
hRXRγ	NM_006917	F	ACCTTGGAGGACCAGGTCATT	TGCTTCGGGCAGGGTGGAATG
		R	GGAGAAAGAGGCAATCAGCAA	
hNur77	NM_002135	F	AGCATTATGGTGTCCGCACAT	TGAGGGCTGCAAGGGCTTCTTCAA
		R	TTGGCGTTTTTCTGCACTGT	
hNurr1	NM_006186	F	TGGGATGGTCAAAGAAGTGGTT	TTTAAAAGGCCGGAGAGGTCGTTTGC
		R	TGGGCTCTTCGGTTTCGA	
hNOR1	NM_006981	F	ATGCCCTTGTCCGAGCTTT	AACACCCAGAGATCTTGATTATTCCAGA
		R	AGCCTGGTCAGTGGGACAGT	
hCAR	NM_005122	F	CACATGGGCACCATGTTTGA	TTTGTGCAGTTTAGGCCTCCAGCTCATCT
		R	AAGGGCTGGTGATGGATGAA	
SXR	NM_003889	F	TCCCCAAATCTGCCGTGTAT	ACAAGGCCACTGGCTATCACTTCAATGTCA
		R	AGCCCTTGCATCCTTCACAT	
hPPARα	NM_005036	F	AGCTCCCGTATCTTTTGTTATGTTG	GTCTGCGCTCCAGAGAGCATCTACTGTCA
		R	TCGATCCGCAGGGTGACT	
hβ-Actin	NM_001101	F	CCTGGCACCCAGCACAAT	ATCAAGATCATTGCTCCTCCTGAGCGC
		R	GCCGATCCACACGGAGTACT	

### Transactivation assay

Cells (1 × 10^5 ^cells per well) were cultured overnight in 24-well plates and then transfected with different plasmids using Lipofectamine (Invitrogen, CA) according to the manufacturer's instruction. A luciferase reporter construct harboring a retinoic acid response element (RARE/DR5) (300 ng) and expression plasmids for RXRα and RARβ (50 ng) (provided by Dr. Ronald Evans, Salk Institute, CA) were used for co-transfection. A Renilla luciferase expression plasmid (10 ng) was also included in co-transfection for normalization of transfection efficiency. Cells were then treated with either DMSO or fenretinide (10 μM) for 48 hours. Fresh medium containing fenretinide was provided every 24 hours. After 48 hours, cells were harvested and firefly and renilla luciferase activities were measured using the Dual Luciferase Reporter Assay System (Promega, Madison, WI) in a luminometer.

### Chromatin immunoprecipitation (ChIP) assay

ChIP was performed using the ChIP assay kit from Upstate (Charlottesville, VA) and antibodies specific for RARβ (1:600, Santa Cruz, Santa Cruz, CA). Control ChIP was performed using a normal rabbit IgG (Santa Cruz, Santa Cruz, CA). The immunoprecipitated DNA fragments were amplified by PCR with primer pairs encompassing the proximal RARE of human RARβ or CYP26A1 gene (RARβ: sense 5'-TGGGTCATTTGAAGGTTAG-3', antisense 5'-GTTCTCGGCATCCCAGTC-3'; CYP26A1: sense 5'-CCGCAATTAAAGATGAACT-3', antisense 5'-TACAGGTCCCAGAGCTTGAT-3').

### siRNA transfection

Scramble siRNA and pre-designed siRNA for human RARβ gene were purchased from Ambion (Austin, TX). Huh-7 cells were transfected with siRNA (10 nM per 1 × 10^5 ^cells) using Lipofectamine™ RNAiMAX Transfection Reagent (Invitrogen, Carlsbad, CA) following the manufacturer's instruction. Cells were harvested 48 hours post-transfection for evaluation of RARβ knockdown efficiency.

### Statistical analysis

Data are presented as mean ± S.E.M. Statistical analysis was performed using Student's *t*-test or one-way ANOVA. Significance was defined by *p *< 0.05.

## Results

### Fenretinide induces apoptosis in Huh-7 and Hep3B cells, but not in HepG2 cells

**Figure 1:**
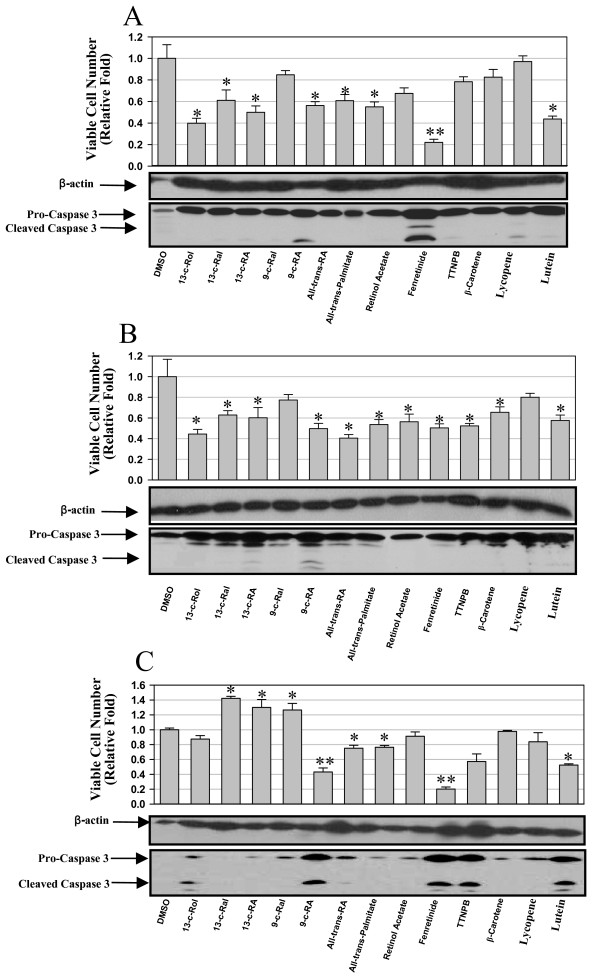
**Retinoids decrease cell viability and induce caspase-3 cleavage in HCC cells**. Huh-7 ***(A)***, HepG2 ***(B)***, and Hep3B cells ***(C) ***were incubated in serum-free medium containing individual retinoids (10 μM) for three days. Viable cells were counted and presented as a relative fold of DMSO treatment. Caspase-3 cleavage in detached cells was determined by Western blot. (* *p *< 0.05, ** *p *< 0.01 compared to DMSO treatment). Data were presented as mean ± S.E.M. Cells were counted in triplicates. Western blot results shown were representative results of two independent experiments.

Several studies have shown that retinoids have anti-proliferation or apoptotic effects in certain cancer cells [4]. To assess the effect of retinoids on HCC cells, we examined cell viability and caspase-3 cleavage induced by individual retinoids in three human HCC cell lines (Figure 1). As an initial screening, 10 μM was used for all the tested retinoids. This dose might be high for certain retinoids, however, besides apoptosis, no obvious cytotoxicity was noted during the 3-day treatment. In Huh-7 cells, nine out of thirteen retinoids decreased cell viability, with fenretinide being the most effective one (79% decrease in cell number compared with DMSO treatment). Fenretinide also induced the strongest caspase-3 cleavage in detached Huh-7 cells and 9-cis retinoic acid caused a modest induction (Figure 1A). In HepG2 cells, although all retinoids examined significantly decreased cell viability, only 9-cis retinoic acid induced weak caspase-3 cleavage (Figure 1B). In Hep3B cells, six out of thirteen retinoids decreased cell viability, whereas another three retinoids increased cell number (Figure 1C). 9-cis retinoic acid, fenretinide, TTNPB, and lutein induced strong caspase-3 cleavage in Hep3B cells (Figure 1C). These findings indicate that fenretinide induces apoptosis in both Huh-7 and Hep3B cells, but not in HepG2 cells.

**Figure 2:**
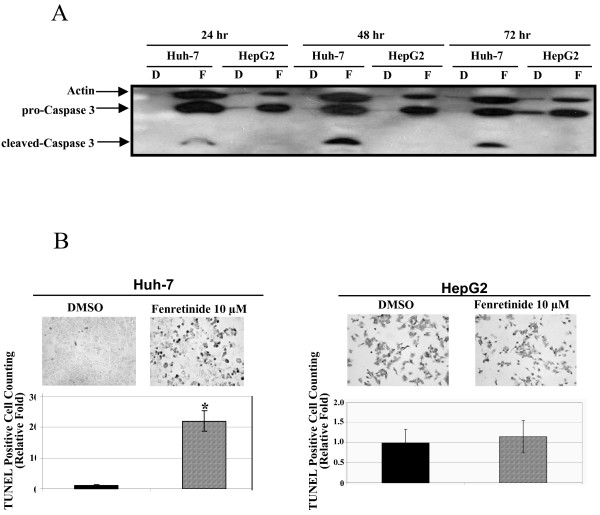
**Fenretinide causes caspase-3 cleavage and DNA double-strand breaks in Huh-7 cells but not in HepG2 cells**. Huh-7 and HepG2 cells were treated with either DMSO or fenretinide and analysed for caspase-3 cleavage by Western blot ***(A) ***and DNA double-strand breaks by TUNEL assay ***(B)***. TUNEL positive counting was presented as a relative fold of DMSO treatment, and the staining was representative result of two independent experiments. Data were shown as mean ± S.E.M. (* *p *< 0.05, ** *p *< 0.01 compared with DMSO treatment).

**Figure 3:**
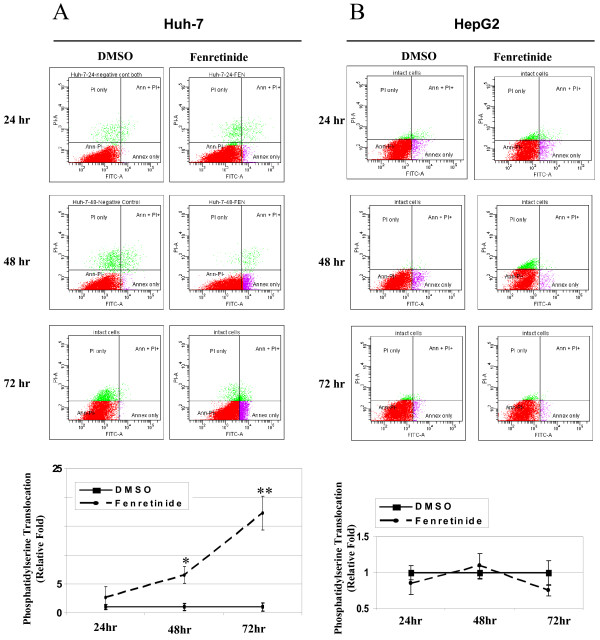
**Fenretinide causes phosphatidylserine translocation in Huh-7 cells but not in HepG2 cells**. Huh-7 ***(A) ***and HepG2 cells ***(B) ***were treated with fenretinide and analysed for phosphatidylserine translocation by flow cytometry analysis. The percentage of cells with phosphatidylserine translocation was presented as a relative fold of DMSO treatment. Flow cytometry data were representative results from three independent experiments. Data were shown as mean ± S.E.M. (* *p *< 0.05, ** *p *< 0.01 compared with DMSO treatment).

To further confirm the differential responses of Huh-7 and HepG2 cells to fenretinide, both cell lines were treated with fenretinide and assessed for caspase-3 cleavage, DNA double-strand breaks, and phosphatidylserine translocation in a time course study. In Huh-7 cells, caspase-3 cleavage was detected at as early as 24 hours after treatment, and the induction was sustained at 48 and 72 hours. In HepG2 cells, however, even after 72 hours treatment, no obvious caspase-3 cleavage was detected (Figure 2A). DNA double-strand breaks, another hallmark of apoptosis, assessed by the TUNEL assay, were detected in Huh-7 cells after treatment (Figure 2B). In contrast, no significant increase in DNA double-strand breaks was detected in HepG2 cells after treatment (Figure 2B). Some background TUNEL staining was detected in HepG2 cells possibly due to the endogenous peroxidase activity. In addition, during apoptosis, membrane lipid phosphatidylserine translocates from the inner leaflet of the plasma membrane to the outer leaflet, resulting in loss of cell membrane asymmetry. Fenretinide induced phosphatidylserine translocation in Huh-7 cells in a time-dependent manner, reaching 17-fold after 72 hours (Figure 3A). However, fenretinide failed to induce such changes in HepG2 cells even after a 3-day treatment (Figure 3B). Taken together, these findings convincingly demonstrate that Huh-7 cells are susceptible to fenretinide-induced apoptosis, but HepG2 cells are resistant.

### High basal and inducibility of RARβ gene expression in HCC cells is associated with their susceptibility to fenretinide-induced apoptosis

**Figure 4:**
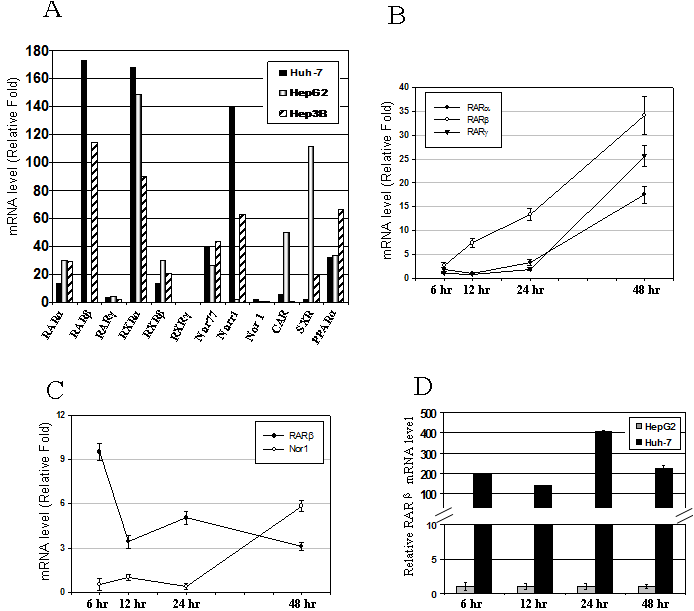
**RARβ expression in HCC cells positively correlates with the susceptibility to fenretinide**. ***(A) ***Basal expression profile of twelve nuclear receptor genes in Huh-7, HepG2, and Hep3B cells. ***(B) ***Fenretinide induced nuclear receptors RARα, β, and γ mRNA in Huh-7 cells. ***(C) ***Fenretinide induced RARβ and Nor1 mRNA in HepG2 cells. ***(D) ***Comparison of RARβ mRNA level between Huh-7 and HepG2 cells after fenretinide treatment (relative fold of RARβ level between Huh-7 and HepG2 cells). Data from two independent experiments were presented as mean ± S.E.M.

To determine whether nuclear receptors mediate the apoptotic effect of fenretinide in HCC cells, the basal mRNA levels of twelve nuclear receptors were assessed by real-time PCR (Figure 4A). Among the three cell lines, Huh-7 cells have the highest basal mRNA levels of RARβ, RXRα, and an orphan nuclear receptor Nurr1 (also known as NR4A2). Hep3B cells have the second highest mRNA levels of RARβ and Nurr1. In contrast, in HepG2 cells, the basal mRNA level of RARβ is undetectable. On the other hand, in HepG2 cells, the basal mRNA levels of SXR (steroid and xenobiotic receptor) and CAR (constitutive androstane receptor), two xenobiotic sensors that mediate many xenobiotic responses, are the highest among the three cell clines.

The regulation of these twelve nuclear receptor genes by fenretinide were then evaluated in Huh-7 and HepG2 cells by real-time PCR. The induction fold was calculated by comparing the mRNA level of each nuclear receptor gene between DMSO and fenretinide treatment at each time point. Only those genes that showed marked changes in expression were presented. In Huh-7 cells, fenretinide caused a continuous induction of RARβ mRNA level (Figure 4B). After 48 hours of treatment, mRNA levels of RARα and γ were also highly induced in Huh-7 cells (Figure 4B). In contrast, a 9-fold induction of RARβ was detected 6 hours after fenretinide treatment in HepG2 cells, and then the induction dropped down to 3–5 fold later on. NOR1 (NR4A3) mRNA was induced 6-fold after 48 hours treatment in HepG2 cells (Figure 4C). Furthermore, by comparing the RARβ mRNA level between Huh-7 and HepG2 cells after fenretinide treatment, our data revealed a dramatic difference in RARβ mRNA level between these two cell lines (Figure 4D). Taken together, these data clearly depict a positive correlation between RARβ mRNA level and susceptibility to fenretinide-induced apoptosis, which suggests that RARβ may play an important role in mediating fenretinide-induced apoptosis in HCC cells.

### Fenretinide activates RXRα/RARβ-mediated pathway

**Figure 5:**
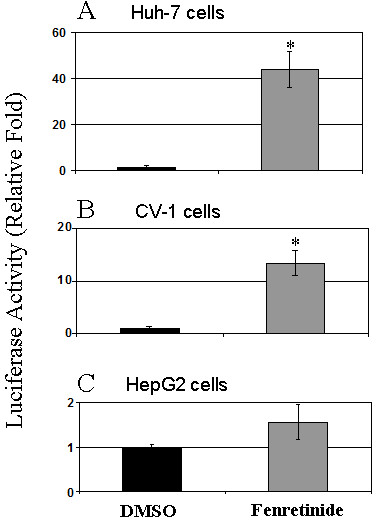
**Fenretinide transactivates RXRα/RARβ-mediated pathway in Huh-7, CV-1 cells, but not in HepG2 cells**. Huh-7 ***(A)***, CV-1 ***(B)***, and HepG2 ***(C) ***cells were transfected with a luciferase reporter harboring RARE (DR-5) and expression plasmids for RXRα and RARβ. Firefly luciferase activity was normalized by co-transfected Renilla luciferase activity. The histograms depict the relative fold of normalized luciferase reporter activity between fenretinide treatment and DMSO treatment. Data from triplicates were presented as mean ± S.E.M.

It is known that RARβ induces it own expression upon stimulation by RAR ligands [10]. Since fenretinide is a synthetic retinoid whether it can directly activate RARβ remains to be tested. So we examined whether fenretinide activates RARβ. We first tested whether fenretinide can activate the RXRα/RARβ-mediated pathway by transactivation assay (Figure 5). Fenretinide caused a marked induction of luciferase activity in Huh-7 cells (43-fold) and in CV-1 cells (13-fold) (Figure 5A and 5B). In contrast, fenretinide did not significantly increase luciferase activity in HepG2 cells (Figure 5C). These data indicate that fenretinide can transactivate RXRα/RARβ-mediated pathway in Huh-7 but not in HepG2 cells. As shown in Figure 4C, a modest induction of RARβ mRNA was seen in HepG2 cells 6 hours after fenretinide treatment, but no sustained induction at 48 hours. Consistently, no significant increase in luciferase activity, which was measured 48 hours after fenretinide treatment, was detected in HepG2 cells.

### Fenretinide increases the transcriptional activity of RARβ in Huh-7 cells

**Figure 6:**
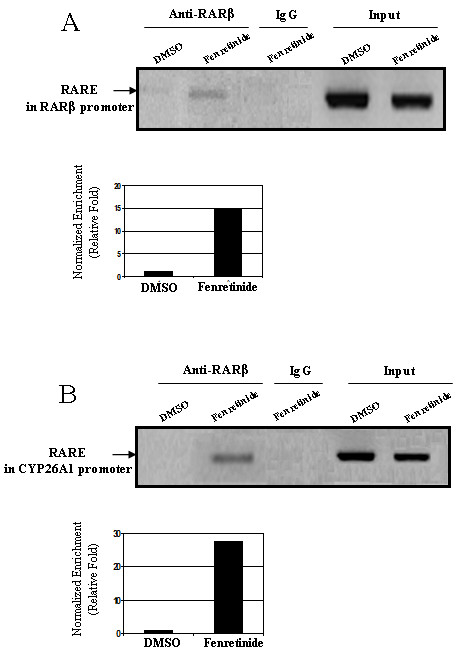
**Fenretinide increases the transcriptional activity of RARβ**. Huh-7 cells were treated with fenretinide for 24 hours followed by ChIP assay. ***(A) ***RARβ and ***(B) ***CYP26A1 promoter fragments (harboring RARE) bound by RARβ were immunoprecipitated with a specific antibody against RARβ and amplified by PCR. Representative PCR results from two independent experiments were shown. The histograms depict the relative fold of PCR band intensity (after normalized to the corresponding input) between fenretinide treatment and DMSO treatment.

The most direct evidence of RARβ activation is the increased binding of RARβ to the response elements in retinoid target genes. It is known that RARβ binds to the RARE in its own promoter upon RA treatment [10,11]. One of the classic RAREs, a 5-bp spaced direct repeat (DR5), was found in the promoter of RARβ [10]. Another well established retinoid target gene is cytochrome P450 26A1 (CYP26A1), an enzyme that catalyzes the breakdown of retinoic acid to more polar metabolites [10]. Two RAREs have been found in the promoter of CYP26A1, one in the proximal region and the other in the distal region [12]. Using chromatin immunoprecipitation (ChIP) assay, the direct binding of RARβ to the RAREs in RARβ and CYP26A1 upon fenretinide treatment was revealed. Fenretinide increased the binding (14-fold) of RARβ to its own promoter compared with DMSO treatment (Figure 6A). An even higher increase (27-fold) of RARβ binding to the CYP26A1 promoter was also noted (Figure 6B). Together, these results demonstrate that fenretinide directly activates RARβ.

### Knockdown of RARβ mRNA expression by siRNA reduces fenretinide-induced apoptosis in Huh-7 cells

**Figure 7:**
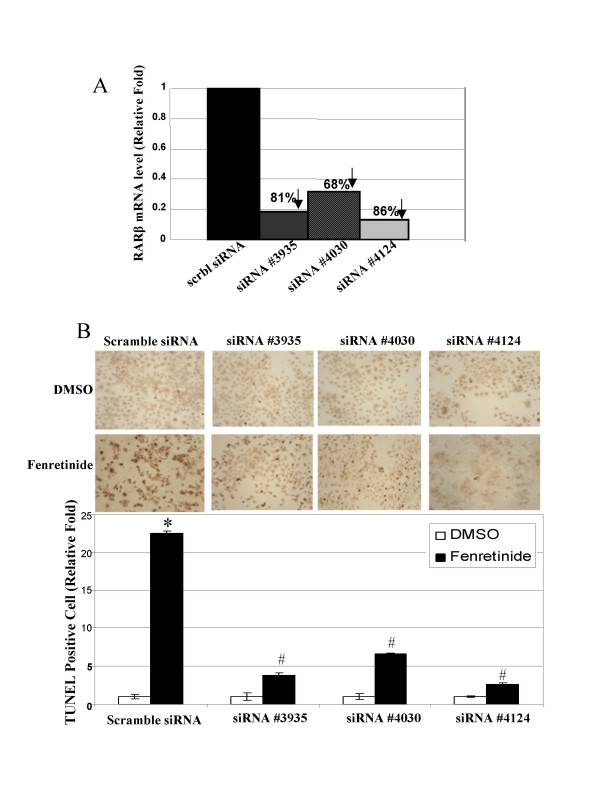
**RARβ knockdown by siRNA diminished fenretinide-induced apoptosis**. ***(A) ***Establishment of RARβ-deficient Huh-7 cells. Silencing of RARβ was achieved by transfecting Huh-7 cells with individual pre-designed RARβ siRNAs (#3935, #4030, and #4124) or scramble siRNA as the negative control. The knockdown efficiency of RARβ (i.e. RARβ mRNA level reduction) by each siRNA was presented as the percentage of RARβ mRNA level in scramble siRNA-transfected cells 48 hours post-transfection. ***(B) ***Scramble or RARβ siRNA transfected Huh-7 cells were treated with either DMSO or fenretinide for 24 hours followed by TUNEL assay. TUNEL staining was representative result from two independent experiments. The histograms depict the relative fold of TUNEL positive cells between fenretinide and DMSO treatment in individual siRNA transfected Huh-7 cells. TUNEL positive cell counting was presented as mean ± S.E.M. from four independent countings (* *p *< 0.05, compared with DMSO treatment of scramble siRNA-transfected Huh-7 cells; ^# ^*p *< 0.05, compared with fenretinide treatment of scramble siRNA-transfected Huh-7 cells).

To determine the role of RARβ in mediating fenretinide-induced apoptosis, the endogenous RARβ mRNA expression in Huh-7 cells was knocked down using siRNA. The knockdown efficiency of RARβ by three sequence-independent siRNA oligonucleotides was evaluated by real-time PCR. Three siRNAs silenced RARβ gene expression to different extents, the most efficient knockdown being 86% (siRNA #4124) compared with scramble siRNA, followed by 81% (siRNA #3935) and 68% (siRNA #4030) (Figure 7A). The apoptotic effect of fenretinide was then evaluated in these RARβ-deficient cells. Our results showed that DNA double-strand breaks induced by fenretinide were markedly reduced in RARβ-deficient Huh-7 cells (Figure 7B). Consistent with RARβ knockdown level, the greatest reduction of apoptosis (88.6%) was seen in the cells with the lowest endogenous RARβ mRNA level (cells transfected by siRNA #4124 with 86% knockdown of RARβ mRNA), followed by 83.1% in cells transfected by siRNA # 3935 with 81% knockdown of RARβ mRNA, and 70.7% in cells transfected by siRNA # 4030 with 68% knockdown of RARβ mRNA. These data clearly demonstrate that fenretinide-induced apoptosis of Huh-7 cells is RARβ dependent.

## Discussion

Retinoids have emerged as important signaling molecules in the regulation of cellular homeostasis. During the past decade, the knowledge on the mechanisms of retinoids action has been greatly expanded due to the discovery and characterization of retinoid receptors and the consensus RAREs in retinoid target genes [13]. Retinoid receptors are ligand-dependent transcription factors that regulate expression of retinoid target genes upon activation [10]. One retinoid receptor, RARβ, has been speculated as a tumor suppressor in several studies. Decreased RARβ expression was found in head and neck squamous cell carcinoma [14], premalignant oral lesions [15], and esophageal squamous cell carcinoma [16]. Suppression of RARβ causes resistance to retinoic acid-associated growth arrest in breast and prostate cancer cells [17] and in F9 teratocarcinoma cells [18]. Induced RARβ expression sensitizes non-small cell lung cancer cells and colorectal cancer cells to the anticancer effects of retinoids [19]. However, how RARβ exerts its role as a tumor suppressor is largely unknown.

In the present study, we identified fenretinide from a panel of retinoids and carotenoids as the most effective one in inducing apoptosis in HCC cells. We further identified RARβ as the key nuclear receptor in mediating the effect of fenretinide. Moreover, evidence from this study clearly demonstrates that fenretinide directly activates RARβ and that RARβ is required for fenretinide-induced apoptosis in Huh-7 cells. Thus, the novel finding of the current study is the identification of a positive correlation between RARβ expression and the susceptibility of HCC cells to fenretinide. This finding suggests a role of RARβ in determining the sensitivity of HCC cells to certain chemotherapeutic agents, which may also hold true for other types of tumor cells.

In fenretinide-resistant HepG2 cells, not only the basal RARβ mRNA level was low, but also the induction of RARβ mRNA by fenretinide was modest and discontinuous. It was known that the promoter of the RARβ gene is frequently hypermethylated in acute myeloid leukemia and cholangiocarcinoma [20,21]. Using DNA methyltransferase inhibitor, the basal RARβ mRNA level in HepG2 cells did not increase (unpublished data) suggesting promoter methylation might not account for the suppressed RARβ mRNA expression in HepG2 cells.

Similar to the expression pattern of RARβ mRNA in HCC cells, the basal expression of Nurr1 is much higher in Huh-7 than in HepG2 cells suggesting Nurr1 might also contribute to the observed differential susceptibility. The basal expression level of Nur77 in Huh-7 and HepG2 cells also correlates with the sensitivity of the cell line to fenretinide-induced apoptosis. Nur77 was shown to enhance RARE activity in transactivation assay [22]. Furthermore, recently studies suggest that Nur77 translocates to mitochondria and interacts with Bcl-2 to promote apoptosis [23,24]. Therefore, the role of Nur77 in fenretinide-induced apoptosis warrants further investigation.

Another major difference regarding the nuclear receptor basal expression pattern is that HepG2 cells express higher basal levels of CAR and SXR mRNA than Huh-7 cells. It is known that activation of CAR or constitutive activation of SXR induces hepatomegaly in mice [25-27]. Whether the high levels of CAR and SXR contribute to the resistance of HepG2 cells to fenretinide-induced apoptosis should be investigated.

Fenretinide seems to be a rather stable compound. Pharmacokinetics studies have shown that fenretinide has a much longer elimination half-life than all-trans and 13-cis retinoic acid [28]. In another study, the tissue concentration of fenretinide and its main metabolite N-(4-methoxyphenyl) retinamide (4-MPR) were determined after a 3-day treatment by HPLC [29]. The data showed that the concentration of fenretinide was 5-fold higher than that of 4-MPR in various mouse tissues including liver. So it is unlikely that the observed apoptotic effect is mediated through fenretinide metabolites. In addition, in the present study, we used cell culture, in which the metabolism rate might not be as efficient as in the liver. To avoid accumulation of the metabolites of fenretinide during treatment, fresh retinoids were provided every 24 hours. So it is highly likely that the observed apoptotic effect was caused by the parent compound rather than the metabolites of fenretinide.

During fenretinide treatment, the other two RARs, RARα and γ, were highly induced after 48 hours (25-fold and 17-fold, respectively) in Huh-7 cells implicating the involvement of these two RARs at the late stage of apoptosis in Huh-7 cells. As some studies have shown, RARα and RARγ may be involved in apoptosis induction in immortalized keratinocytes and leukemia cells [30,31]. In HepG2 cells, however, NOR1 (also known as NR4A3), was induced 6-fold after 48 hours. This induction may contribute to the resistance of HepG2 cells to fenretinide as NOR1 has been suggested to have pro-survival functions in some cell types [32].

Another novel finding is the direct activation of RARβ by fenretinide. It has been shown that fenretinide induces apoptosis in many types of cancer cells including neuroblastoma cells, breast, lung, head and neck, cervical and ovarian cancer cells [8,33,34]. However, the underlying mechanisms are poorly understood. Some studies suggest that the effects of fenretinide are mediated through reactive oxygen species (ROS) and caspase-3 [35], whereas other studies indicate the involvement of ceramide [36] and the NF-κB pathway [37]. Both retinoid receptor dependent and independent mechanisms have been proposed for fenretinide anticancer effects [34]. Our results obtained from transactivation assay and ChIP assay clearly demonstrate that fenretinide directly activates RARβ in Huh-7 cells. Knockdown of RARβ mRNA expression by siRNA provides a direct proof that RARβ is required for fenretinide-induced apoptosis. To the best of our knowledge, this is the first study to report that nuclear receptor RARβ mediates the apoptotic effect of fenretinide in HCC cells. Our findings strongly suggest a potential role of RARβ as a tumor suppressor by mediating the signals of certain chemotherapeutic agents. However, there are still unbridged gaps between RARβ activation and apoptosis execution. Exploration of RARβ target genes will provide helpful insights into these molecular links.

## Conclusion

Our findings reveal that endogenous expression of retinoids receptor RARβ gene determines the susceptibility of HCC cells to fenretinide-induced apoptosis. Our results demonstrate fenretinide directly activates RARβ and induces RARβ-dependent apoptosis in Huh-7 cells. These findings suggest a novel role of RARβ as a tumor suppressor by mediating the signals of certain chemotherapeutic agents.

## Abbreviations

ChIP, chromatin immunoprecipitation assay; HCC, hepatocellular carcinoma; RA, retinoic acid; RAR, retinoic acid receptor; RARE, retinoic acid response element; RXR, retinoid × receptor.

## Competing interests

The author(s) declare that they have no competing interests.

## Authors' contributions

PB carried out all the experiments, participated in data analysis, and drafted the manuscript. YW conceived the study, participated in experiment design and data analysis, and revised the manuscript. Both authors read and approved the final manuscript.

## Pre-publication history

The pre-publication history for this paper can be accessed here:


